# Mucus Containing Cystic Lesions “Mucocele” of the Appendix: The Unresolved Issues

**DOI:** 10.1155/2015/139461

**Published:** 2015-03-23

**Authors:** Mohammad Ezzedien Rabie, Mubarak Al Shraim, Mohammad Saad Al Skaini, Saad Alqahtani, Ismail El Hakeem, Abdulla Saad Al Qahtani, Tarek Malatani, Abduelah Hummadi

**Affiliations:** ^1^Department of Surgery, Armed Forces Hospital, Southern Region, Khamis Mushait, Saudi Arabia; ^2^Department of Pathology, College of Medicine, King Khalid University, Abha, Saudi Arabia; ^3^Department of Radiology, Armed Forces Hospital, Southern Region, Khamis Mushait, Saudi Arabia; ^4^Department of Surgery, College of Medicine, Umm Alqura University, Saudi Arabia

## Abstract

*Background*. Mucocele of the appendix is a rare condition, the pathological classification and management strategy of which have not been standardized yet. *Aim*. To report on our management of appendiceal mucocele, highlighting the pitfalls and possible means for avoiding them. *Materials and Methods*. Our registries were reviewed to retrieve cases of appendiceal mucocele, encountered in the period from July 2008 to May 2013. *Results*. We had 9 cases, three males and sex females, with a median age of 62 years. Abdominal ultrasound (US) and computerized axial tomography scan (CT) suspected the diagnosis in only one case each. Open appendectomy was done in two cases of mucinous cystadenoma with no further surgery performed, despite the positive margin in one. Laparoscopic appendectomy was done in three cases: mucinous cystadenoma in one case which needed no further surgery, mucinous cystadenocarcinoma with pseudomyxoma peritonei in another, and low grade mucinous tumour in a third case, and all needed subsequent right hemicolectomy. Exploratory laparotomy was done in three cases: of these, synchronous right hemicolectomy was done in one case of mucinous cystadenoma/?mucinous tumour of uncertain malignant potential; in the other two cases, appendectomy only was done for mucinous hyperplasia with carcinoid tumour of the appendix in one case and mucinous cystadenoma/?mucinous tumour of uncertain malignant potential in another. The 9th case was discovered upon laparoscopy for cholecystectomy; when pseudomyxoma peritonei arising from an appendiceal mucocele was found, laparoscopic appendectomy with peritoneal biopsy was then performed instead. Histopathologic diagnostic uncertainty was present in two cases of mucinous cystadenoma where mucinous tumour of uncertain malignant potential was an alternative possibility. Perioperative colonoscopy was performed in only one case and our follow-up programme was defective, with the longest period being 180 days. *Conclusion*. Mucocele of the appendix should be considered in the differential diagnosis of cystic lesions in the right lower abdomen. Owing to its rarity, it continues to intrigue the surgeon as well as the radiologist and pathologist alike. For mucinous cystadenocarcinoma, right hemicolectomy is usually needed, whereas for hyperplasia and cystadenoma, appendectomy usually suffices if the resection margins are free. For mucinous tumours of uncertain malignant potential and low grade mucinous tumours as well as pseudomyxoma peritonei, the decision is not as simple. As for laparoscopic surgery, no solid proof exists with or against its safety. Although not yet standardized, perioperative colonoscopy and regular follow-up to detect early recurrences should probably be part of the management plan.

## 1. Introduction

The term mucocele of the appendix was coined by Karl Freiherr von Rokitansky in 1842 [1–3]. It is a morphologic term, describing the transformation of the appendix into a mucus-filled sac, regardless of the aetiology. The condition has an incidence ranging from 0.07% [4] to 0.63% [5], and it affects both sexes between the 5th and 7th decades of life [6].

On the histopathological level, the diagnosis is not always straightforward and the terminology has not been completely settled [7]. On the other hand, pseudomyxoma peritonei, which signifies the presence of pools of mucin and mucin secreting cells within the peritoneal cavity, is a feared complication of appendicular mucocele [8]. The term was first introduced by Werth in 1884 [9, 10] and, likewise, several categories exist, with inconsistent characterization and terminology [11, 12]. In advanced cases, the peritoneal cavity becomes filled with mucinous material, and the condition is termed jelly belly syndrome [11]. On the clinical level and due to the rarity of the condition, no official guidelines exist and the management is based on personal as well as departmental experience, in addition to case reports and small case series.

## 2. Materials and Methods

The registries of the surgical wards, operation theatre, and histopathology department were reviewed to retrieve cases of appendiceal mucocele, encountered in the period from July 2008 to May 2013. The patients' medical records were accessed to extract relevant demographic, clinical, radiological, and histopathological data. The management of each patient was recorded as well as his condition on follow-up. To estimate the prevalence of the condition in our locality, the number of appendectomies performed in the same period was calculated.

## 3. Results

**Figure 1:**
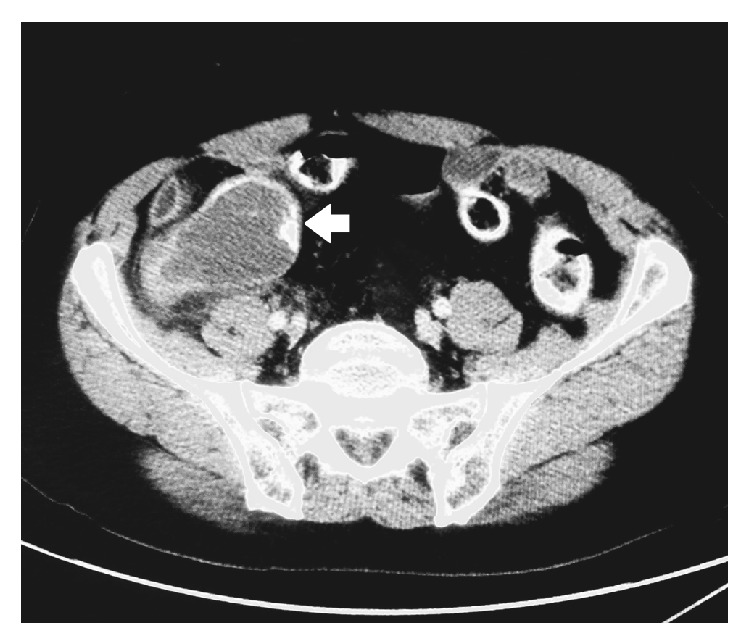
Mucocele of the appendix with wall calcification (white arrow).

We were able to retrieve nine cases, three males and six females, with an incidence of 0.5% and a median age at presentation of 62 years (range 26–88). Five patients presented acutely, while the other four had chronic presentation. The main complaint was abdominal pain in all cases. The patients' demographics and admission diagnosis are shown in Tables 1 and 2 The median white blood count was 9600/*μ*L (range 2500–17700, reference range 4.000–10.000) and leucocytosis was found in two cases, while the median C reactive protein, measured in five cases, was 83.3 mg/L (range 20.6–161.5, reference range < 10 mg/L). Preoperative US scan was done in seven cases, and the diagnosis was suspected in only one case, whereas CT scan was done in eight cases and again the diagnosis was suspected in only one. Radiologic findings on CT scan were as follows: a cystic/tubular structure was found in eight cases. It was hypodense in 7 cases and hyperdense in only one case. All cases showed varying degree of wall enhancement, and only three showed punctuate, curvilinear, or linear calcification (Figure 1). In only one case was fine needle aspiration (FNA) considered, but fortunately the procedure was not carried out after considering the possibility of appendicular mucocele. The initial operation, histopathologic diagnosis, margin status, and further operation are shown in Table 3.

After initial surgery, colonoscopy followed by right hemicolectomy was done in one case of appendicular mass, proved to be mucinous cystadenocarcinoma on histopathology (the 7th patient), whereas postoperative follow-up CT scan was performed in only two cases (in the second and third patients) after 43 and 180 days after surgery, respectively. Only one case (the 8th patient) was referred to the oncologist.

**Figure 2:**
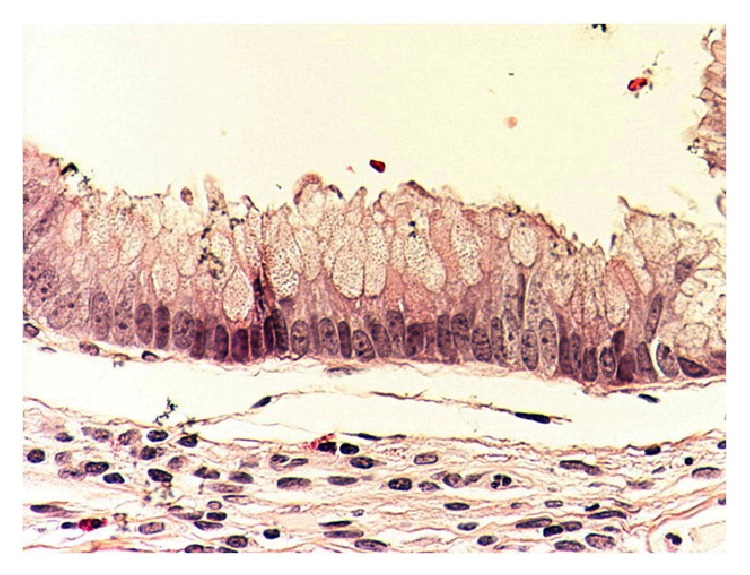
Photomicrograph showing appendiceal mucosal mucinous hyperplasia with no cytological epithelial atypia (original magnification ×20, hematoxylin and eosin stain).

**Figure 3:**
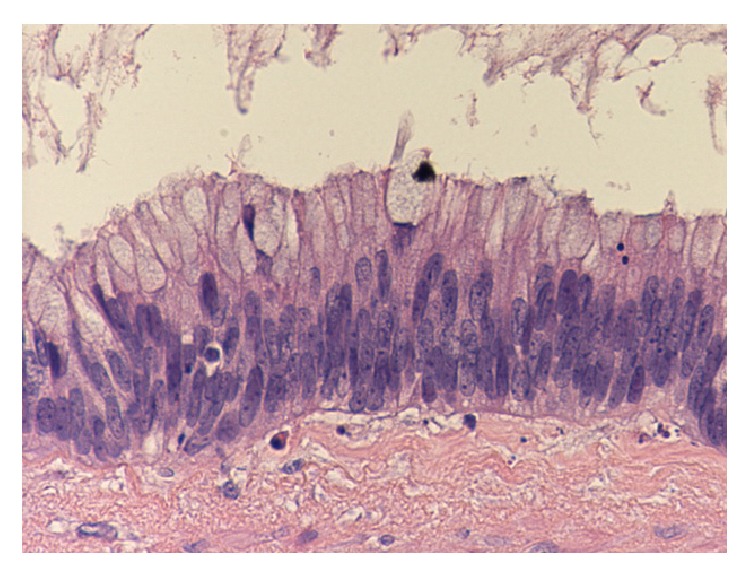
Photomicrograph showing mucinous cystadenoma with mild pleomorphism and nuclear atypia (original magnification ×20, hematoxylin and eosin stain).

**Figure 4:**
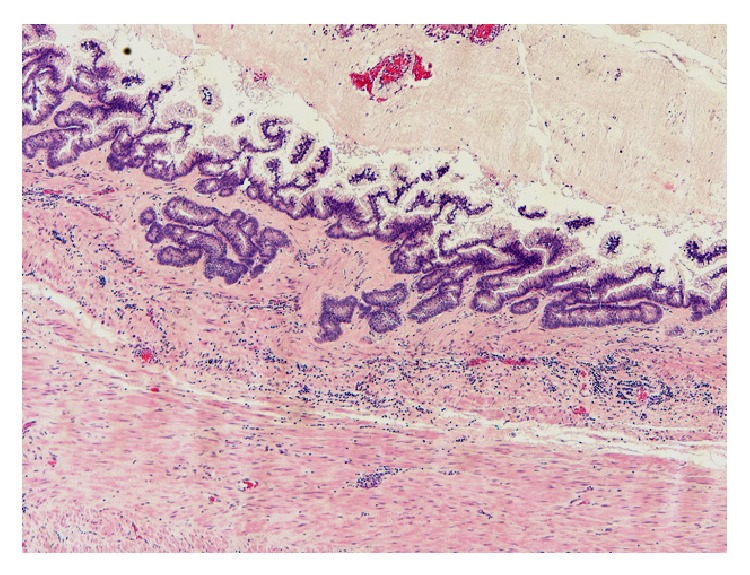
Photomicrograph showing low grade mucinous cystadenocarcinoma with complex cribriform glands invading the appendiceal wall (original magnification ×4, hematoxylin and eosin stain).

**Figure 5:**
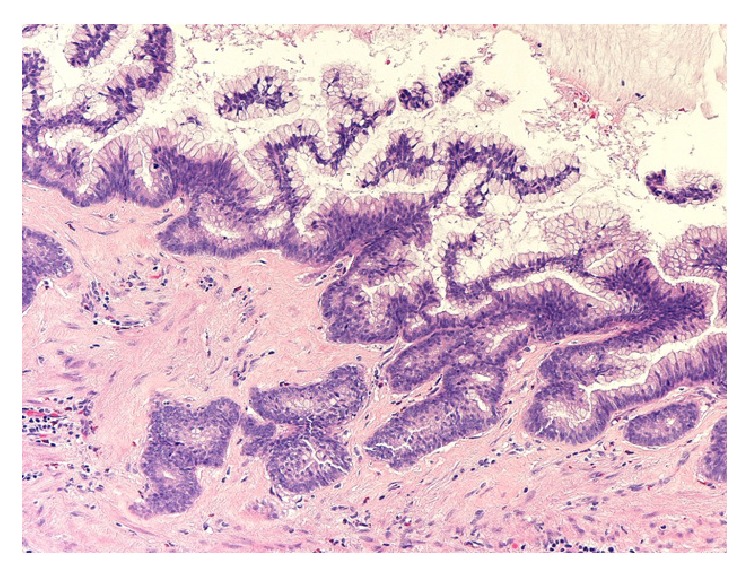
Photomicrograph showing low grade mucinous cystadenocarcinoma with nuclear pleomorphism and frequent mitotic figures (original magnification ×20, hematoxylin and eosin stain).

**Figure 6:**
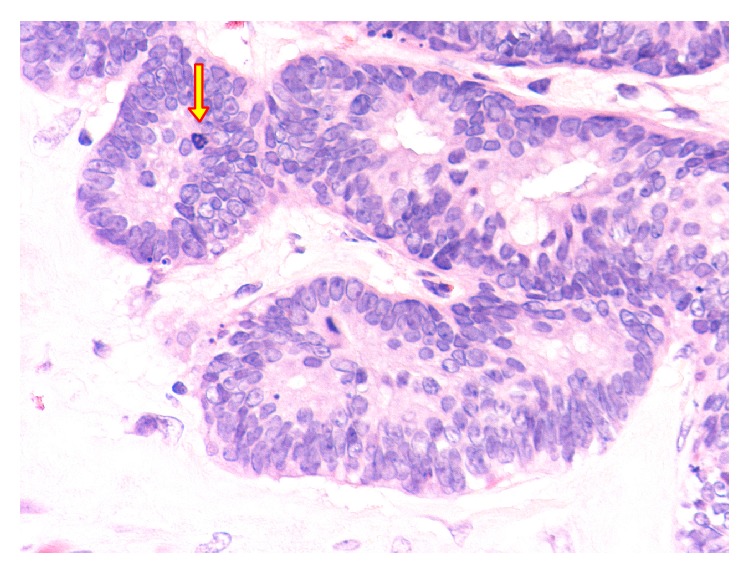
Photomicrograph showing low grade mucinous cystadenocarcinoma. The tumor cells with a full-thickness nuclear stratification and increased mitotic figures (arrow) (original magnification ×40, hematoxylin and eosin stain).

**Figure 7:**
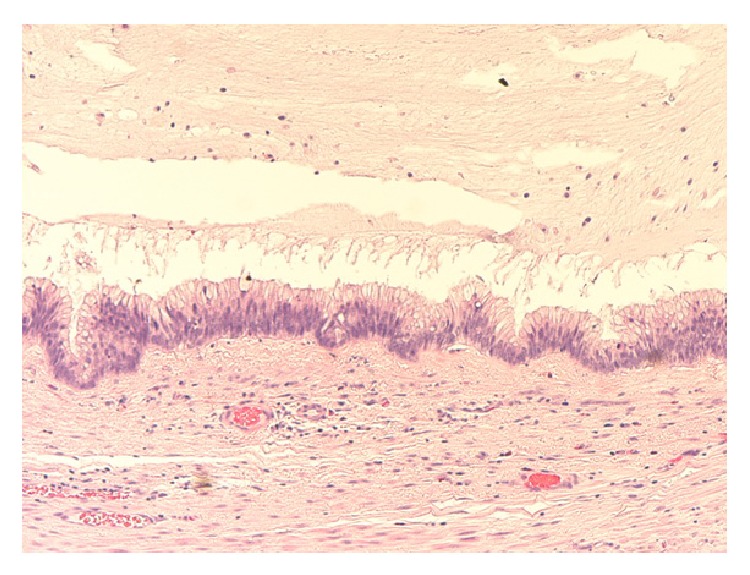
Photomicrograph showing mucinous tumour of uncertain malignant potential with mild pleomorphism and mild nuclear atypia. No invasion of the wall by neoplastic epithelium (original magnification ×10, hematoxylin and eosin stain).

*Histopathology.* Mucinous cystadenoma was found in five cases (in two of them mucinous tumour of uncertain malignant potential was an alternative diagnosis), mucinous cystadenocarcinoma with pseudomyxoma peritonei in two cases, mucinous hyperplasia with carcinoid tumour of the appendix in one case, and low grade mucinous tumour in another case (Figures 2, 3, 4, 5, 6, and 7). In three cases, an associated acute appendicitis was found.

Here we demonstrate two representative cases at the extreme ends of the scale.

*First Case.* A 62-year-old female, with past history of hypertension, diabetes mellitus, rheumatoid arthritis, osteoporosis, left hemithyroidectomy, umbilical hernia repair, and cholecystectomy, presented with abdominal pain and constipation for one month. Apart from mild tenderness in the right iliac fossa, her general and abdominal examinations were normal. Her investigations showed normal liver and renal functions, and her blood picture was normal except for a low white cell count of 2500/*μ*L.

**Figure 8:**
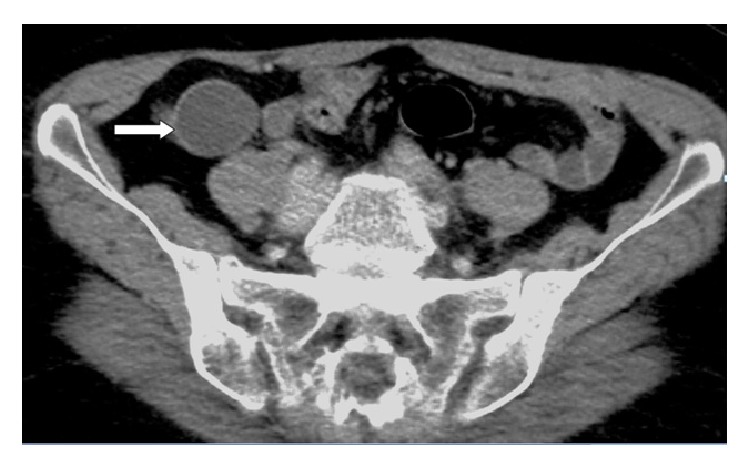
CT scan of the abdomen showing a mass in the right iliac fossa misdiagnosed as appendicular mass, white arrow (first case).

**Figure 9:**
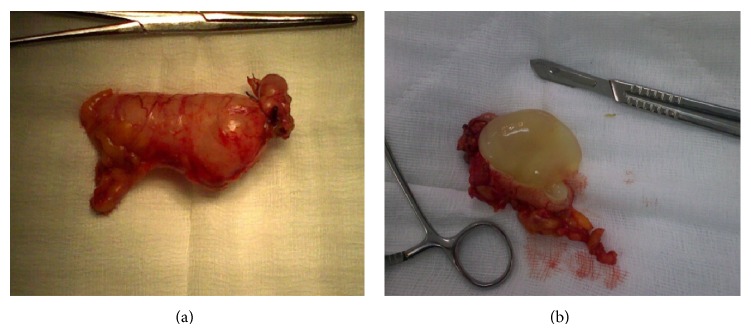
((a) and (b)) The excised appendix distended with mucous (first case).

US/CT scan showed a mass in the right iliac fossa, for which the patient was admitted as a case of appendicular mass (Figure 8). The following day, laparoscopic appendectomy was performed. During the procedure, the appendix was seen distended in a saccular form, suggestive of a mucocele, with no involvement of the surrounding tissues and no mucoid material in the peritoneal cavity (Figure 9). Straightforward appendectomy was done and the specimen was removed intact in an endopouch. The postoperative recovery was uneventful and the patient was discharged for follow-up. Histopathology showed mucinous cystadenoma of the appendix with no involvement of the base. A couple of days later, she appeared in the clinic in good condition and was discharged from further follow-up.

**Figure 10:**
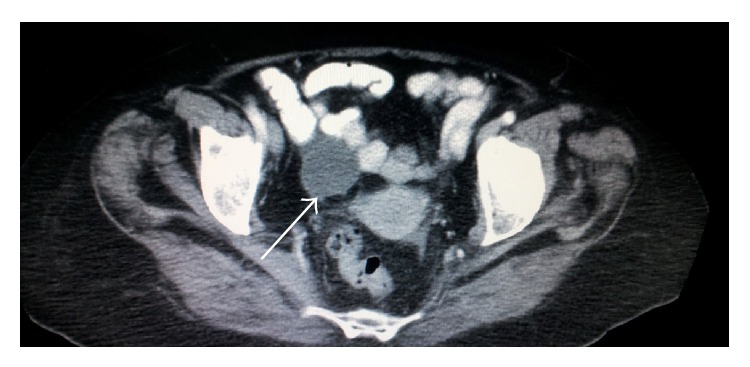
CT scan showing a swelling in the right iliac fossa (white arrow), misdiagnosed as ovarian cyst (second case).

**Figure 11:**
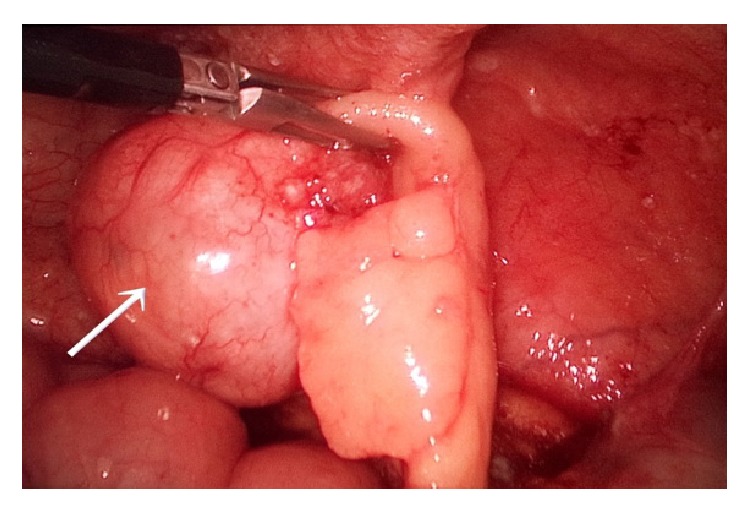
Swelling at the distal part of the appendix, white arrow-laparoscopic view (second case).

**Figure 12:**
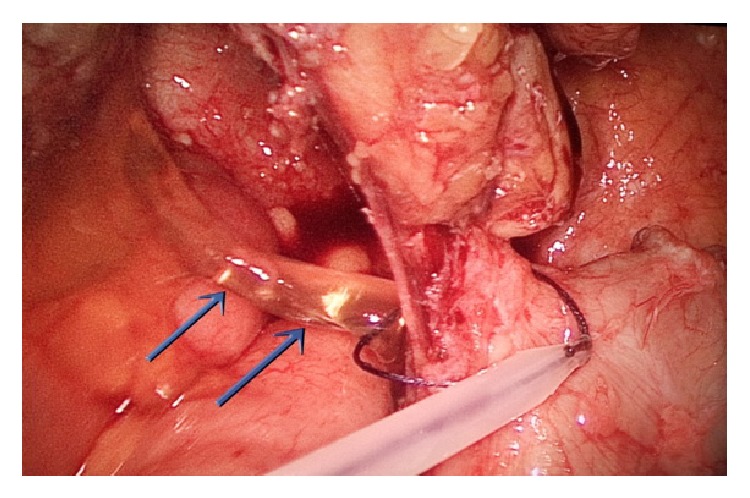
Oozing of mucus from the surface of the appendix (blue arrows), with the endoloop being applied to the base of the appendix, laparoscopic view (second case).

*Second Case.* A 60-year-old female, with no past medical history, presented with vague abdominal pain and US evidence of gallstones. On examination, her abdomen was soft with no tenderness and there was an unusually firm paraumbilical hernia. Her blood works were within normal, and due to the atypical character of pain CT scan was requested, which showed gallstones, an umbilical facial defect, and a right ovarian cyst (Figure 10). The patient was posted for laparoscopic cholecystectomy and paraumbilical hernia repair. During dissection of the hernia for port insertion, two small gelatinous nodules came into view and were excised. After inserting the camera, the peritoneal cavity appeared studded with gelatinous nodules of varying sizes (Figures 11 and 12). Two more ports were inserted to explore the abdomen and there was a collection of gelatinous material in the right iliac fossa with a lemon-sized swelling at the distal half of the appendix, which poured gelatinous material from its surface. The appendix was excised laparoscopically, retrieved in an endobag, and sent for histopathology along with few nodules from the peritoneal surface. The hernia was repaired anatomically and the procedure was terminated. The histopathology showed mucinous cystadenocarcinoma of the appendix with pseudomyxoma peritonei. She was then referred to the oncologist for further management.

## 4. Discussion

In the opinion of some authors, the term “mucocele” should be abandoned in favour of a more specific pathological term [8]. In our opinion, the term should be retained for some reasons, with the knowledge that it comprises different pathological categories. Firstly, being deeply rooted in the literature, its meaning is understandable for all. Secondly, it is a simple descriptive term, which enables the surgeon or radiologist to report on the lesion when encountered, before any pathological characterization is obtained.

Pathologically, the condition may be classified into neoplastic and nonneoplastic variants. The neoplastic variant results from overproduction of mucus by a mucinous tumour of the appendix. It includes three main categories: mucosal hyperplasia, cystadenoma, and cystadenocarcinoma [8, 13, 14]. In mucosal hyperplasia, there is no epithelia atypia, whereas in mucinous cystadenoma, there is some degree of epithelial atypia and, additionally, acellular mucus may be present in the periappendiceal region or free in the peritoneal cavity. These two variants are benign and simple appendectomy is curative. Although pseudomyxoma peritonei did not follow these two categories in Higa series [13], it was observed to follow them in other series [15]. On the other hand, mucinous cystadenocarcinoma is characterized by the presence of stromal invasion by malignant glands and/or the presence of mucus and mucus secreting cells in the peritoneal cavity [8, 13]. Relatively, recently, intermediate grades between cystadenoma and cystadenocarcinoma, namely, mucinous tumour of uncertain malignant potential and low grade mucinous neoplasm, have been added [7, 16]. Moreover, other terms do exist. Furthermore, some authors divide low grade mucinous neoplasm into mucinous neoplasm with low risk of recurrence and mucinous neoplasm with high risk of recurrence, with each subtype having a different clinical behaviour. Mucinous neoplasm with low risk of recurrence is an appendiceal tumour with histologic features of mucinous adenoma but with extra appendiceal acellular mucin. On the other hand, mucinous neoplasm with high risk of recurrence has the morphological features of mucinous adenoma but with clear evidence of neoplastic epithelial spread beyond the muscularis propria [17]. This controversy in the pathologic terminology can give rise to a clinical dilemma in terms of the management and follow-up plans.

Contrary to the aforementioned categories, the nonneoplastic variant results from chronic insidious obstruction of the appendiceal lumen by any process other than mucinous neoplasia [7, 8, 18]. This leads to retention of mucus behind the obstruction and finally its seepage to the outside as the intraluminal pressure increases. It encompasses different types according to the obstructive lesion and was thus termed inflammatory, obstructive, simple mucocele or retention cyst of the appendix [19]. Obstruction by a faecolith and endometriosis of the appendix are included here [8, 18, 20].

The most common mode of presentation of appendiceal mucocele is right lower quadrant pain simulating acute appendicitis [21–23], with an incidence of 45% of cases [6, 24]. In this series, it was seen in 4 out of 9 cases.

Other presenting modes include lower abdominal mass, bowel obstruction, anaemia, weight loss, and chronic abdominal pain [6, 24]. Bowel obstruction may be brought about by a variety of ways [25], including extrinsic compression [26] or torsion of the mucocele [27, 28], thus bringing the condition to light, as was observed in one of our patients who presented with bowel obstruction.

A preoperative diagnosis is obviously needed in order to plan the procedure and avoid rupture of the mucocele, with subsequent development of pseudomyxoma peritonei. This preemption is the exception rather than the rule and a preoperative diagnosis is far less likely, as was seen in our work. In this context, we failed to obtain a radiologic diagnosis in almost all cases. Moreover, ancillary laboratory investigations are also unhelpful. In this series, although leucocytosis and raised C reactive protein were found in several patients, being nonspecific and inconsistent, their utility, if any, is negligible [29].

Certain radiologic features of appendicular mucocele have been identified [25, 30]. In our series, US scan raised the possibility of mucocele in one out of seven cases, while (CT) scan raised this possibility in one out of eight cases, highlighting our inability to reach a preoperative diagnosis in the vast majority of cases. Additionally, appendiceal mucocele was mistaken for an adnexal mass in one of our patients, a mistake which has been previously reported [31, 32].

It is always tempting to acquire histologic or cytologic needle biopsy for a newly discovered abdominal mass or cyst. For obvious reasons, this should never be attempted once the diagnosis of mucocele is suspected [33]. This scenario was narrowly averted in one patient, when FNA was planned, but fortunately refuted after considering the possibility of mucocele.

Although laparoscopic appendectomy for appendiceal mucocele had been followed by wide dissemination of mucinous implants on the peritoneal surface several months later [34], gentle handling of the appendix, avoiding its rupture or its direct contact with the other viscera or the parities, can prevent this serious complication, and currently laparoscopic appendectomy is being increasingly performed [35–37]. In our series, laparoscopic appendectomy was done in four cases, with no adverse events noted. Obviously, the specimen has to be extracted in a retrieval bag. Despite that, in the absence of a solid proof through randomized controlled trials, the safety of laparoscopic surgery remains putative at best, and more research efforts are obviously needed.

The association between mucocele of the appendix on one side and synchronous or metachronous colorectal tumours and ovarian mucinous tumours on the other side is well documented [6, 8, 13, 38]. For this reason, colonoscopy and appropriate radiology in the pre- or postoperative periods, as applicable, as well as thorough exploration during surgery, are indicated. Unfortunately, colonoscopy was performed only once in this series. Additionally, oncologist referral and long-term CT follow-up scan were sparsely done in our series. In this regard, our practice is not much different from that of others, as these issues are rarely performed, and this certainly needs to be addressed. In this regard, it has been suggested that no follow-up is needed for cases of simple and hyperplastic mucocele, whereas those due to cystadenoma require follow-up as for colonic adenoma and those due to cystadenocarcinoma should be followed up as for colonic adenocarcinoma [19].

The presence of periappendiceal mucoid material does not always mean malignancy, as it has been seen with hyperplasia and cystadenoma [6]. However, pseudomyxoma peritonei may complicate mucinous cystadenoma, in addition to cystadenocarcinoma [15]. In our series, pseudomyxoma peritonei was only associated with cystadenocarcinoma.

While surgery is the only known potentially curative treatment, currently, there is no consensus regarding the optimal management. However, the extent of surgery, which ranges from appendectomy to right hemicolectomy, depends on several factors. The size of the tumour, its location within the appendix, involvement of the cecum and ileum, presence of mucus collections, the safety margin, involvement of lymph nodes, and the final histology [6, 24] are the determining factors. Moreover, with synchronous colonic tumours, the extent of surgery should obviously encompass the colonic pathology, regardless of the characters of the mucocele itself [39].

We reviewed the literature and found that all work in this area is composed mostly of case reports and not many case series. We also reviewed the Cochrane Library to explore the presence of published clinical trials or systematic reviews, utilizing the terms “mucocele of the appendix” and “appendicular mucocele,” but our search yielded no result. We searched the PubMed for publications using the same terms, and the majority of papers were case reports, with only 65 case series over the period from 1919 till 2014, which represents a modest number relative to this long duration. Moreover, we searched the National Comprehensive Cancer Network (NCCN), National Institute of Clinical Excellence (NICE), and the Cochrane Collaboration for published guidelines, and we did not find any. We also reviewed the uptodate.com website and confirmed the absence of guidelines published by authoritative bodies.

However, a useful algorithm has been suggested by Filho and associates [19]. When the appendiceal base is not involved, appendectomy is done with excision of all the mesoappendiceal fat and contained lymph nodes. If the base is involved, typhlectomy, using the linear cutting stabler (GIA), or partial right hemicolectomy, is done well away from the appendix base. Frozen section is performed, and if no malignancy is found, as in cases of hyperplasia and cystadenoma, the operation is terminated. On the other hand, proved malignancy, that is, cystadenocarcinoma, necessitates an oncologic right hemicolectomy. If frozen section is not available, as is usually the case in the unplanned emergency setting, the permanent paraffin section is awaited for and again oncologic right hemicolectomy is performed if malignancy is discovered. Likewise, appendectomy only suffices for cases of endometriosis, carcinoid and adenocarcinoid tumours of the appendix provided that the histology is favourable, the base is uninvolved, the tumour is less than 2 cm, and the lymph nodes are negative. If these conditions are not satisfied, right hemicolectomy should be performed [40, 41]. Our surgeries were concordant with the above-mentioned lines, except in one case of cystadenoma with positive resection margins, where no further surgery was done (Table 3).

In this series, the difficulty in arriving at the correct histopathologic diagnosis in some cases was evident, where cystadenoma versus mucinous tumour of uncertain malignant potential was considered in two patients (Table 3). This uncertainty has probably no clinical implications, as the resection margins were free in both cases with no mucinous material found in the peritoneal cavity. As these tumours behave in a benign or low grade fashion [42], appendectomy alone may suffice, if the previous two stipulations are fulfilled. Alternatively, right hemicolectomy may be performed [43], although there is no solid proof to its superiority to appendectomy alone, under the aforementioned circumstances [42].

On the other hand, Ronnett and associates classified pseudomyxoma peritonei into three categories: (1) disseminated peritoneal adenomucinosis (DPAM), where there is abundant mucin and scanty simple mucinous cells with little atypia and mitotic activity, (2) peritoneal mucinous carcinomatosis (PMCA), where the cellular and architectural features of carcinoma exist, and (3) peritoneal mucinous carcinomatosis with intermediate or discordant features (PMCA-I/D), where intermediate features exist [15]. The prognosis is best for DPAM followed by PMCA-I/D and is worst for PMCA. The prognostic implications of this classification have been validated in a recent work, where it was found that the 10-year survival rate for the three categories is 65.0%, 28.0%, and 14.0%, respectively, with the younger age carrying a poorer prognosis [44].

Like the term “mucocele,” some authors discourage using the term pseudomyxoma peritonei, with the pretext that it is a morphologic rather than an accurate pathologic term [44]. Our opinion is on the contrary for the same reasons given before to retain the term “mucocele” in common use. The management of pseudomyxoma peritonei entails cytoreductive surgery as well as chemotherapy. During surgery, peritonectomy, which is a complex and lengthy procedure, taking an average of 10 hours, is performed. It is combined with intraperitoneal and, recently, intraoperative intraperitoneal heated chemotherapy [11, 12]. Both procedures have high morbidity and mortality rates, and, for this reason, centralization of the management to few centres has been called for. This aims to deepen the experience with a rarely encountered disease and thus improve the outcome of therapy. In this series, two cases of pseudomyxoma peritonei associated with cystadenocarcinoma of the appendix were encountered. The first case received right hemicolectomy, from which she convalesced. Unfortunately, five weeks after surgery, she developed inferior myocardial infarct with left ventricular extension. Therefore, no further oncologic referral was done. The second patient was admitted for cholecystectomy, and mucocele of the appendix with pseudomyxoma peritonei was found. Laparoscopic appendectomy and peritoneal biopsy were done and the patient was referred to the oncologist for subsequent management.

## 5. Conclusion

Mucocele of the appendix should be considered in the differential diagnosis of cystic lesions in the right lower abdomen. Owing to its rarity, it continues to intrigue the surgeon as well as the radiologist and pathologist alike. For mucinous cystadenocarcinoma, right hemicolectomy is usually needed, whereas for hyperplasia and cystadenoma, appendectomy usually suffices if the resection margins are free. For mucinous tumours of uncertain malignant potential and low grade mucinous tumours as well as pseudomyxoma peritonei, the decision is not as simple. As for laparoscopic surgery, no solid proof exists with or against its safety. Although not yet standardized, perioperative colonoscopy and regular follow-up to detect early recurrences should probably be part of the management plan.

## Figures and Tables

**Table 1:** Patients' demographics and admission diagnosis.

Serial number	Age	Sex	Admission diagnosis
1	80	♀	Abdominal pain FI
2	58	♀	Appendicular abscess?
3	62	♀	Appendicular mass
4	48	♂	Acute appendicitis
5	80	♀	Large bowel obstruction
6	88	♂	Abdominal pain FI
7	59	♀	Abdominal pain FI
8	60	♀	Chronic calculous cholecystitis
9	26	♂	Acute appendicitis

**Table 2:** Admission diagnosis and its frequency.

Admission diagnosis		Frequency
Acute appendicitis		4
Simple	2	
Appendicular abscess?	1	
Appendicular mass	1	
Abdominal pain for investigations		3
Large bowel obstruction		1
Chronic calculous cholecystitis, paraumbilical hernia, and Rt. ovarian cyst		1

Total		9

**Table 3:** Initial operation and histopathologic diagnosis, margin status, and further operation.

Serial	Initial operation	Histopathology	Resection margin	Further operation
1	Open appendectomy	Mucinous cystadenoma with acute appendicitis	Positive	No

2	Exploratory laparotomy and right hemicolectomy	Mucinous cystadenoma with acute and chronic inflammation/mucinous tumour of uncertain malignant potential?	Free	No

3	Laparoscopic appendectomy^**^	Mucinous cystadenoma	Free	No

4	Open appendectomy	Mucinous cystadenoma	Free	No

5	Exploratory laparotomy with appendectomy	Mucinous hyperplasia with carcinoid tumour of the appendix and necrotizing hepatic granulomas	Free	No

6	Exploratory laparotomy with appendectomy	Mucinous cystadenoma with acute and chronic inflammation/mucinous tumour of uncertain malignant potential?	Free	No

7	Laparoscopic appendectomy^**^	Mucinous cystadenocarcinoma with peritoneal and omental deposits/pseudomyxoma peritonei	Not documented^*^	Rt. hemicolectomy

8	Laparoscopic cholecystectomy converted to laparoscopic appendectomy and peritoneal masses biopsy^**^	Mucinous cystadenocarcinoma with peritoneal and omental deposits/pseudomyxoma peritonei	Not documented^*^	No

9	Laparoscopic appendectomy^**^	Low grade mucinous tumour	Free	Rt. hemicolectomy

^*^The presence of peritoneal and omental deposits with pseudomyxoma peritonei invalidates the need for a free resection margin.

^**^Retrieval bag was used to extract the specimen.
